# A clustering approach for detecting implausible observation values in electronic health records data

**DOI:** 10.1186/s12911-019-0852-6

**Published:** 2019-07-23

**Authors:** Hossein Estiri, Jeffrey G. Klann, Shawn N. Murphy

**Affiliations:** 1 Laboratory of Computer Science, Massachusetts General Hospital, 50 Staniford Street, Suite 750, Boston, MA 02114 USA; 2 Research Information Science and Computing, Partners HealthCare, Charlestown, MA USA; 3 Department of Neurology, Massachusetts General Hospital, Boston, MA USA

**Keywords:** Unsupervised clustering, Implausible observations, Data quality, Electronic health records, Informatics applications, Anomaly detection

## Abstract

**Background:**

Identifying implausible clinical observations (e.g., laboratory test and vital sign values) in Electronic Health Record (EHR) data using rule-based procedures is challenging. Anomaly/outlier detection methods can be applied as an alternative algorithmic approach to flagging such implausible values in EHRs.

**Methods:**

The primary objectives of this research were to develop and test an unsupervised clustering-based anomaly/outlier detection approach for detecting implausible observations in EHR data as an alternative algorithmic solution to the existing procedures. Our approach is built upon two underlying hypotheses that, (i) when there are large number of observations, implausible records should be sparse, and therefore (ii) if these data are clustered properly, clusters with sparse populations should represent implausible observations. To test these hypotheses, we applied an unsupervised clustering algorithm to EHR observation data on 50 laboratory tests from Partners HealthCare. We tested different specifications of the clustering approach and computed confusion matrix indices against a set of silver-standard plausibility thresholds. We compared the results from the proposed approach with conventional anomaly detection (CAD) approaches, including standard deviation and Mahalanobis distance.

**Results:**

We found that the clustering approach produced results with exceptional specificity and high sensitivity. Compared with the conventional anomaly detection approaches, our proposed clustering approach resulted in significantly smaller number of false positive cases.

**Conclusion:**

Our contributions include (i) a clustering approach for identifying implausible EHR observations, (ii) evidence that implausible observations are sparse in EHR laboratory test results, (iii) a parallel implementation of the clustering approach on i2b2 star schema, and (3) a set of silver-standard plausibility thresholds for 50 laboratory tests that can be used in other studies for validation. The proposed algorithmic solution can augment human decisions to improve data quality. Therefore, a workflow is needed to complement the algorithm’s job and initiate necessary actions that need to be taken in order to improve the quality of data.

**Electronic supplementary material:**

The online version of this article (10.1186/s12911-019-0852-6) contains supplementary material, which is available to authorized users.

## Background

### Implausible observations in electronic health records

Data stored in Electronic Health Records (EHR) offer promising opportunities to advance healthcare research, delivery, and policy. Provision of these opportunities is contingent upon high quality data for secondary use. Data quality concerns, however, have hampered secondary use of EHR data [1, 2]. The increasing throughput of EHR data constantly deposited into clinical data research networks have cultivated new opportunities for utilizing innovative statistical learning methods to improve quality.

Plausibility is a dimension of data quality that represents whether EHR data values are “believable”. [3]. It is quite possible to witness an implausible observation (IO) in EHR data, such as a negative A1c value. An IO is extremely unlikely to signify a fact about a patient and may represent an underlying data quality issue. Detecting such IOs in EHR data are difficult for two reasons. First, gold standards are not always available for all clinical observations to set cut-off thresholds for an implausible observation. Second, even when gold standards are present, detection of out-of-range observations requires limits to be determined for each type of observation, potentially customized at each institution (due to variance in normal ranges). This rule-based approach is becoming increasingly impractical given the every-increasing diversity of observations, ontologies and measurement units across institutions.

With the abundance of unlabeled data (e.g., vital signs) in EHR repositories, unsupervised learning can offer solutions for characterizing clinical observations into meaningful sub-groups. In unsupervised learning, the machine develops a formal framework to build representations of the input data to facilitate further prediction and/or decision making [4]. We focus on records of laboratory results and vital signs in the EHR. The variance and variety of these domains make it particularly difficult to manually assign rules for identifying implausible values. Conceptualizing implausible laboratory test results and vital sign values in EHRs as outliers, we propose and evaluate the feasibility of an unsupervised clustering approach for detecting implausible EHR vital sign and laboratory test values in large scale clinical data warehouses.

### Outlier detection

Outlier detection has been widely applied in medical informatics for addressing different issues, such as detecting unusual patient-management actions in ICU [5], deriving workflow consensus from multiple clinical activity logs [6], characterizing critical conditions in patients undergoing cardiac surgery [7], discovering unusual patient management [8], alert firing within Clinical Decision Support Systems [9], finding clinical decision support malfunctions [10], identifying high performers in hypoglycemia safety in diabetic patients [11], and classifying the influence factor in diabetes symptoms [12].

In outlier detection, the objective is discriminate non-conforming observations from a larger group of observations that conform to similar attributes (inliers) [13]. Due to discrepancies in defining outlyingness, various outlier detection techniques have been proposed [14]. Both parametric (model-based) and non-parametric (model-free) approaches are common for detecting outliers. Non-parametric approaches do not assume a-priori statistical models and therefore can be more suitable for clinical data that are often irregularly sampled. Distance- and density-based techniques utilize different proximity (distance) measures to identify outlying observations. Distance-based methods use local distance measures, such as the Mahalanobis distance, to identify outliers based on distance from the nearest neighbors [15, 16]. Although these methods are popular and often scale to large data [17], their performance decreases in high dimensional spaces [14]. Moreover, the performance of distance-based methods relies on a distance threshold for identifying outliers, and in our experience (including the results in this study), can lead to high false positive rates when applied to human biological data.

Density-based techniques, in contrast, assume that the data density around a inlier data point is similar to the density around its neighbors. Clustering techniques can utilize a density-based approach, e.g. to identify sparse clusters for outlier detection [18]. In fact, clustering techniques can combine attributes from both the distance-based and the density-based methods. We will use a hybrid hierarchical-k-means clustering method in this study, in order to perform a more meaningful detection of outlying observations from human biological data based on both distance and sparsity.

### The hierarchical K-means clustering method

The goal in unsupervised clustering is to partition data points into clusters with small pairwise dissimilarities [19, 20]. K-means [21] is one of the most popular unsupervised clustering algorithms [19], for its simplicity and efficiency. It is a top-down algorithm that aims to minimize the distortion measure by iteratively assigning data points to cluster centroids to meet a convergence criterion [4, 19]. K-means is sensitive to outliers [22, 23]. Although this property is often considered a weakness, sensitivity to outliers makes K-means a good algorithm for our purpose of identifying rare events in clinical observations.

For a vector of observations *x*(1), *x*(2), …, *x*(*n*), where *x*(*i*) ∈*ℝ*^*n*^, the K-means algorithm aims to predict *k* centroids and assign the data points to each centroid to form clusters *C*(*i*), while minimizing the average within-cluster dissimilarity, as follows:

1. Randomly initialize *k* cluster centroids *μ*(1), …, *μ*(*k*)
2. For a given cluster assignment *C*, iterate the following steps until the cluster assignments do not change:
  - $$ C(i):= \underset{k}{\mathrm{argmin}}\parallel x(i)-\mu (k){\parallel}^2. $$
  - $$ \mu (k):= \frac{\sum_{i=1}^nI\left\{C(i)=k\right\}x(i)}{\sum_{i=1}^nI\left\{C(i)=k\right\}}. $$

This specification makes K-means’ performance dependent on initialization of a few hyper-parameters, including the number of clusters and the initial cluster centroids [19, 20]. The dependency of K-means on random initialization of the cluster centers often results in the algorithm’s performance being unreliable, when the number of iterations are small [23]. In contrast to K-means, hierarchical clustering is a bottom-up or agglomerative approach that does not require knowing the number of clusters in advance. As a result, its performance is not dependent on random initialization of *k* cluster centroids. However, compared with the K-means algorithm, hierarchical clustering algorithm is more computationally intensive.

Hybrid hierarchical-k-means (HK-means) clustering [23] combines the strengths of hierarchical clustering in initializing the cluster centroids, and improves efficiency of the K-means algorithm. The HK-means algorithm relaxes the dependency of K-means algorithm on random initialization of cluster centroids by first computing hierarchical clustering, cutting the tree in *k* clusters, computing the centroids for each cluster, and then using the centroids as the initial cluster centers to run K-means. [23]. This hybrid approach accelerates the K-means procedure, and thereby, improves the overall learning [23].

Performance of the HK-means algorithm still depends on approximation of the number of clusters, *k*. Initializing the number of clusters for the algorithm is a challenging problem, for which a number of ad-hoc (or intuition-based) solutions are available [24–26]. Unfortunately, most of the available solutions do not scale up to large datasets.

## Methods

### Approach

We propose an unsupervised approach using the hierarchical-k-means method to detect outlier values in lab tests and vital sign values based on two hypotheses. First, we hypothesize (hypothesis #1) that implausible observations – that are not due to systematic human error – must be sparse (infrequent) in datasets that contain large amounts of data. For example, we do not expect to frequently see a blood pressure record of 1090/80 in an EHR repository. Given ontological harmony, we can use data from EHRs to compare an individual data point with a large set of similar data points and identify implausible observations. Therefore, we also hypothesize (hypothesis #2) that a well-specified unsupervised clustering algorithm should be able to partition clinical observations into meaningful clusters, from which we can extract clusters with sparse populations (very small members of data points) as implausible observations.

**Fig. 1:**
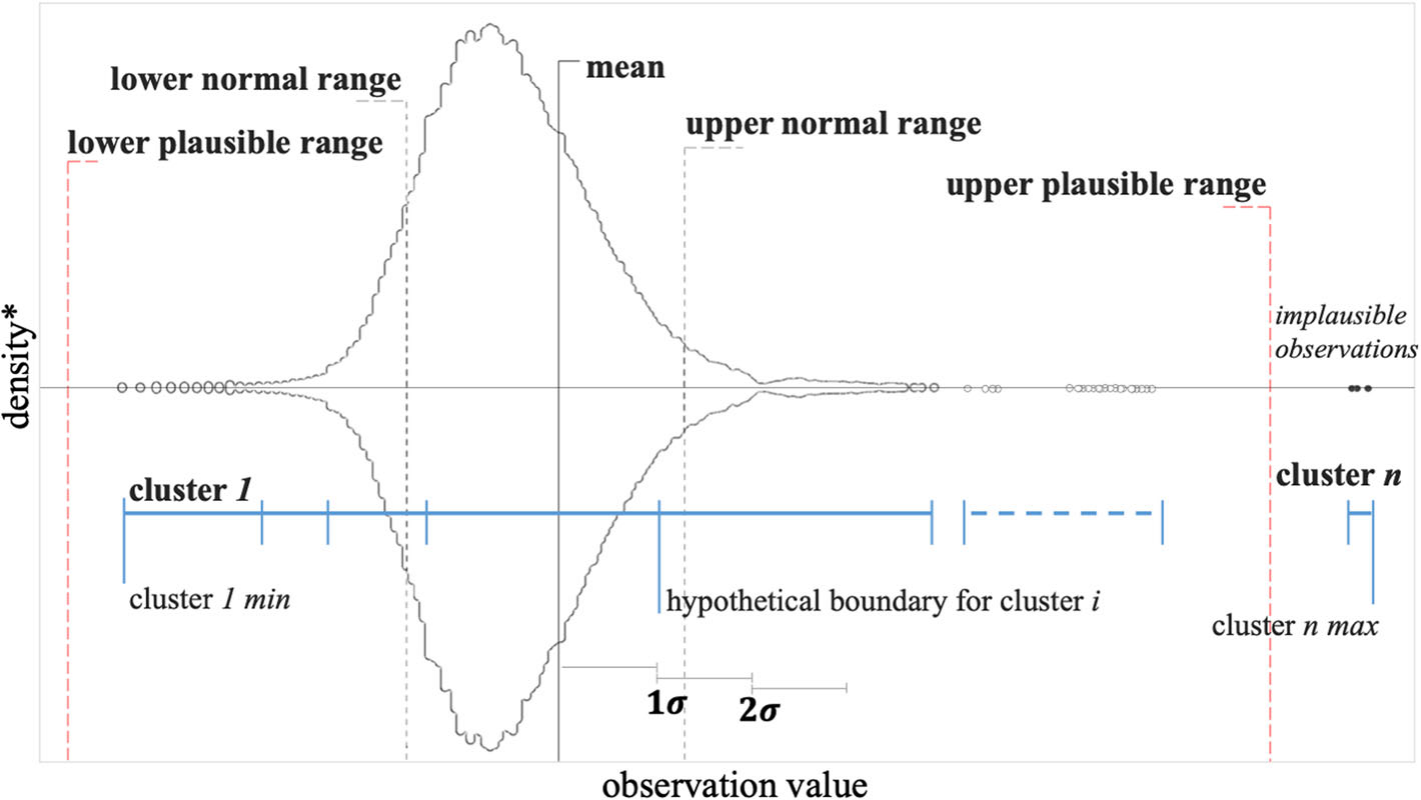
Detecting implausible observations through unsupervised clustering. * density values are mirrored around 0 for visualization purpose

Figure 1 illustrates the rationale behind our second hypothesis. The bell-shaped curve in Fig. 1 is a probability density function for a laboratory result in EHR – extracted and visually modified from Cholesterol in HDL. The hypothetical thresholds for normal ranges and implausible values are delineated on the plot. An unsupervised clustering algorithm partitions the lab values into *n* clusters, each of which embrace a number of data points. We can obtain the number of data points in each cluster and flag clusters with population smaller than a certain threshold as anomalies. Unlike conventional methods such as using standard deviation to identify anomalies, the clustering approach would provide a more flexible solution that is density-based. In addition, the clustering approach should be able to detect implausible observations regardless of their values.

To test these hypotheses, we implemented the clustering solution on EHR observation data from a portion of the Research Patient Data Registry (RPDR) from Partners HealthCare [27], using a hybrid hierarchical K-means clustering algorithm. Specifically, we used *kluster*, an R package that uses iterative sampling to produce scalable and efficient cluster number approximation solutions in large datasets [28]. We set different ratios for flagging a cluster as anomalous. To compare the results against conventional methods, we also performed anomaly detection using conventional approaches (CAD) using standard deviation and Mahalanobis distance [29, 30] with different configurations for each.

We measured the performance for each algorithm against a set of silver-standard high and low implausible thresholds that we manually curated based on literature search and expert judgment, and validated using data distributions in RPDR – Table of silver standards is available in the Appendix (Table 4).

### Data

The data used in this study contained ~ 720 million rows of medical record data representing 50 laboratory observations and a small set of common vital signs, all with distinct Logical Observation Identifiers Names and Codes (LOINC) codes. This data represented contained all results for these 55 observations in the RPDR for patients at Massachusetts General Hospital and Brigham and Women’s Hospital between 2001 and 2018. The RPDR derives these data from various clinical systems, such as the institutional Clinical Data Repository (CDR), the Longitudinal Medical Record (LMR) system, and EpicCare. The lab data are assigned LOINC codes by the Clinical Data Repository team. Otherwise, the data are not modified from the source system before being provided to researchers. On average, each observation type contained more than 14 million data points (range 45,000 to > 69 million). These data are made available for the Accessible Research Commons for Health (ARCH), a 12-site PCORnet Clinical Data Research Network (CDRN).

### Implementation

**Fig. 2:**
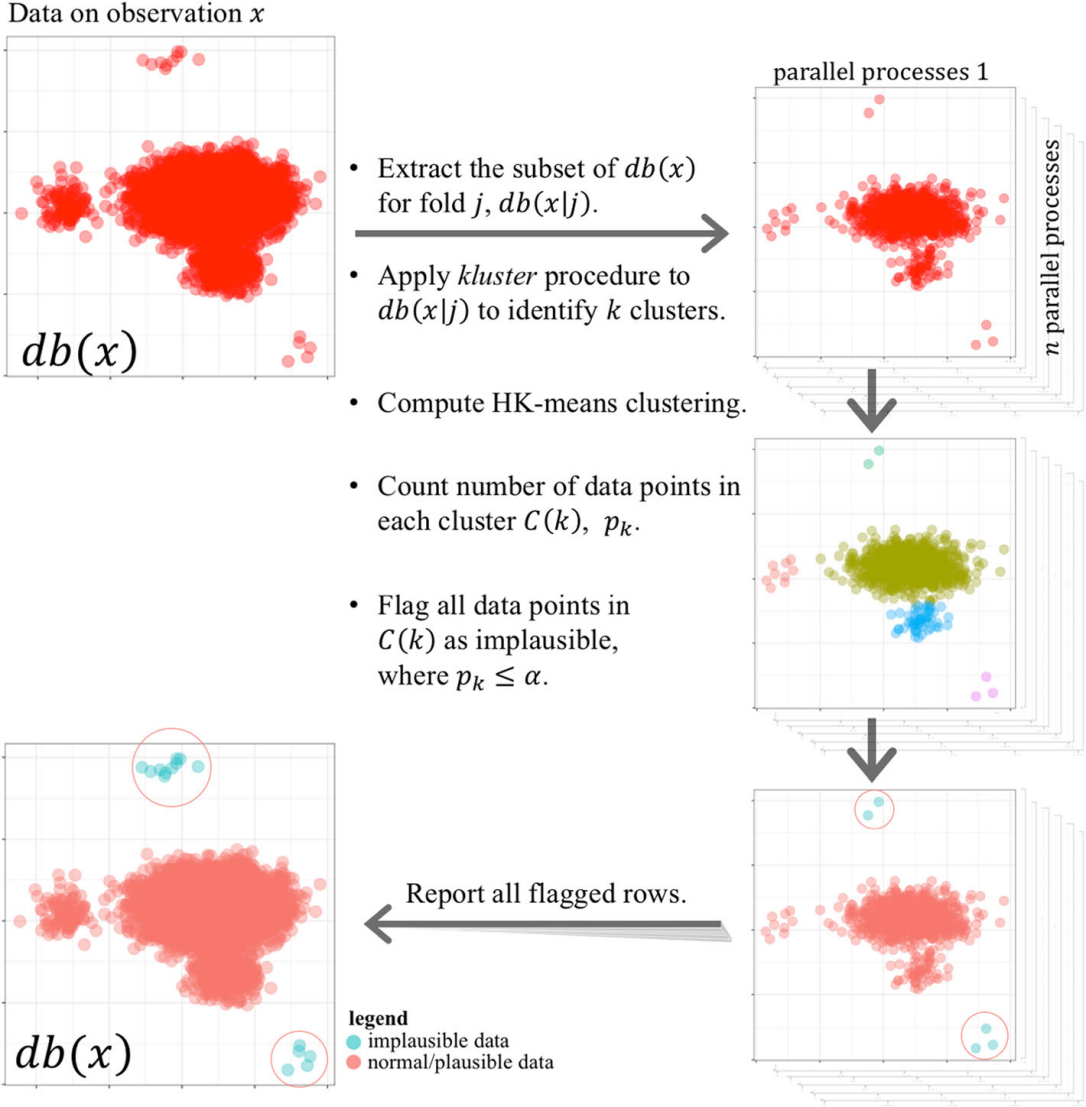
Parallel implementation of the clustering solution for identifying implausible EHR observations

Due to the size of data for each type of lab or vital sign observations, we parallelized the implementation of our clustering solution for identifying the implausible lab observations. Through the parallelization, we also incorporate a hyperparameter that represent the sparsity assumption in the approach. We used R statistical language and high performance computing cluster – provided by the Partners HealthCare’s Enterprise Research Infrastructure & Services – to implement the algorithm, described in following steps (also Fig. 2):

1. Extract data on observation *x*, stored as *db*(*x*), from RPDR.
2. Shuffle *db*(*x*) randomly to avoid any specific sorting for parallelization.
3. Break *db*(*x*) into *j* folds such that each fold has ***n*** (or fewer) data points.
  We controlled the threshold for flagging clusters as implausible ($\boldsymbol{\alpha} =\frac{\mathbf{1}}{\boldsymbol{n}}$) through the selection of the number of data points for parallelization. We evaluated the performance of the proposed methodology using eight thresholds for ***α***: 1/500, 1/1000, 1/2000, 1/3000, 1/4000, 1/5000, 1/6000, and 1/10,000.
4. Begin parallel computing:
  Extract the subset of *db*(*x*) for fold *j*, *db*(*x*| *j*).
  Scale *db*(*x*| *j*) and transform the values to the 3rd power – to focus on distribution tails.
  Apply *kluster* procedure [28]. to *db*(*x*| *j*) to identify *k* clusters.
  Compute HK-means clustering and assign data points to clusters cluster *C*(1), …, *C*(*k*).
  Count number of data points in each cluster *C*(*k*), *p*_*k*_.
  Flag all data points in *C*(*k*) as implausible, where *p*_*k*_ ≤ *α*.
5. Produce a report containing all flagged rows.

To measure performance, we compute confusion matrix indices, including false/true positives, false/true negatives, sensitivity, specificity, and fallout across the eight *α* s. True positive in the confusion matrix represents the number of truly implausible observations (as identified from the silver-standard labels) that were also identified by the clustering approach as implausible.

We also evaluated whether our proposed clustering approach performed better than conventional anomaly detection (CAD) methods, namely identifying implausible values using standard deviation and anomaly detection with Mahalanobis distance (Table 1).

**Table 1:**
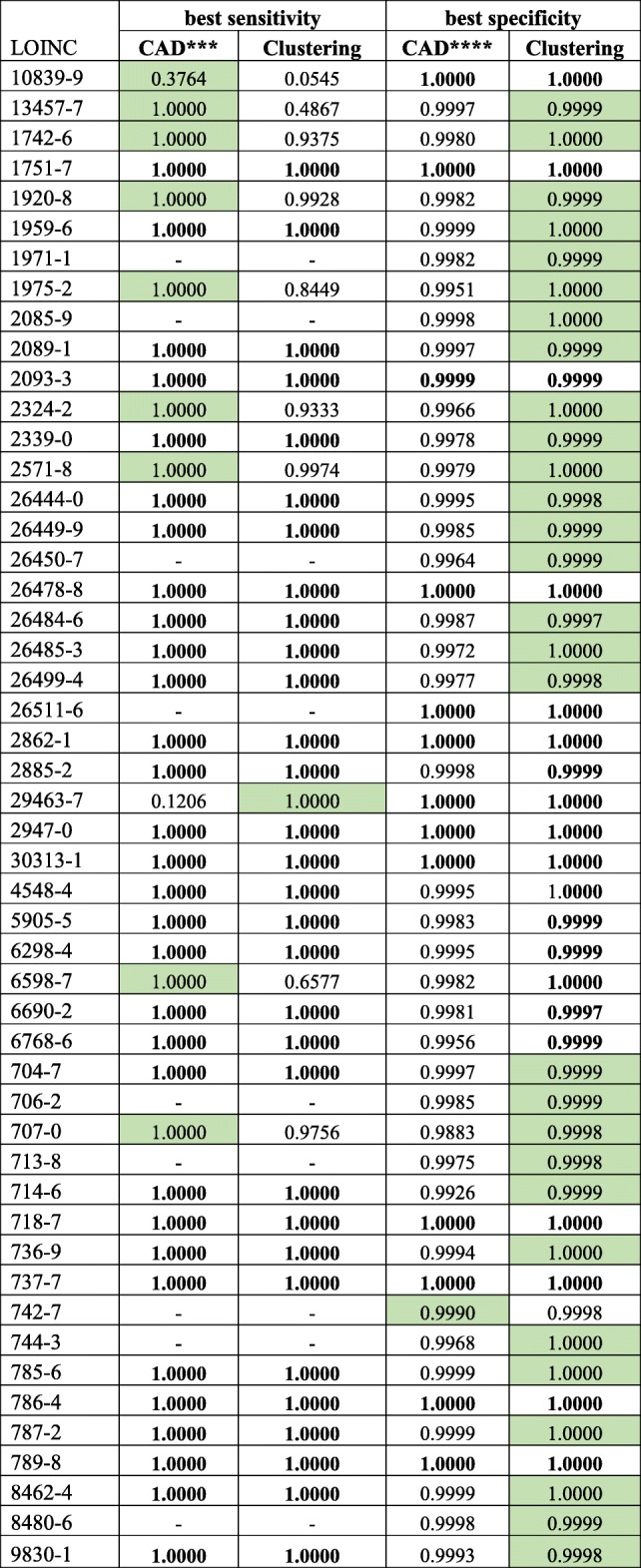
Specificity from the clustering approach for identifying implausible lab observations in EHRs

* Columns represent different thresholds, a, for flagging a cluster as implausible

** Best performances are highlighted

## Results

Sensitivity and specificity metrics for all implementations can be seen in Tables 2 and 3. Here we present a summary, beginning with specificity (i.e., how many data points identified as plausible by the clustering approach are truly plausible).

**Table 2:**
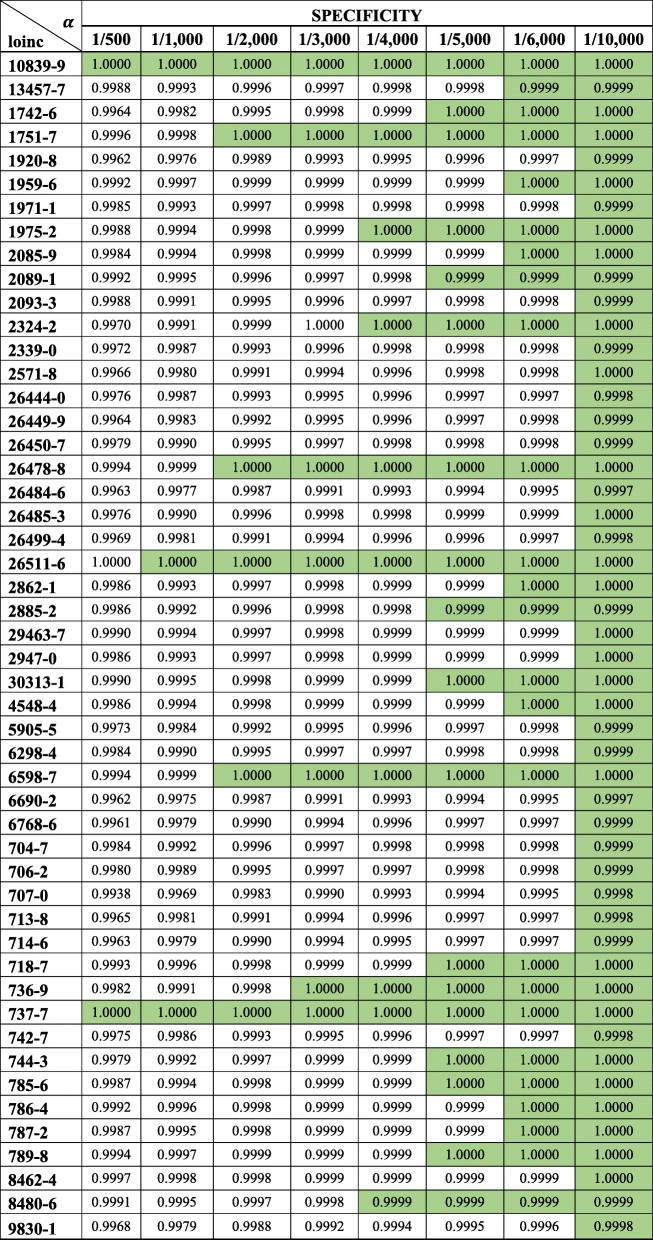
Sensitivity from the clustering approach for identifying implausible lab observations in EHRs * Columns represent different thresholds, a for flagging a cluster as implausible.

** Best performances are highlighted

**Table 3:**
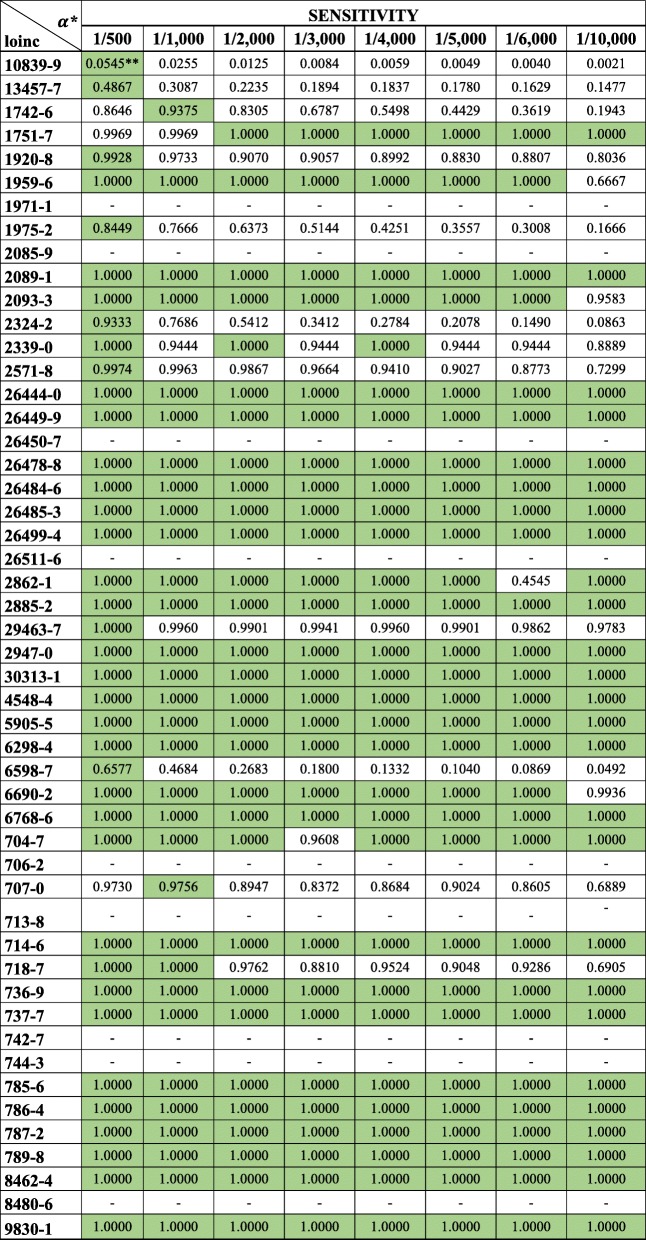
Comparing performance between conventional anomaly detection (CAD) and the proposed clustering approach

* best performances are highlighted

** ties are in Bold

*** best sensitivity among CAD methods was obtained from applying Mahalanobis Distances and 3.717526 (sqrt of 13.82) as critical value

**** best specificity among CAD methods was obtained from using 6 standard deviations as threshold for identifying outliers

### Specificity

**Fig. 3:**
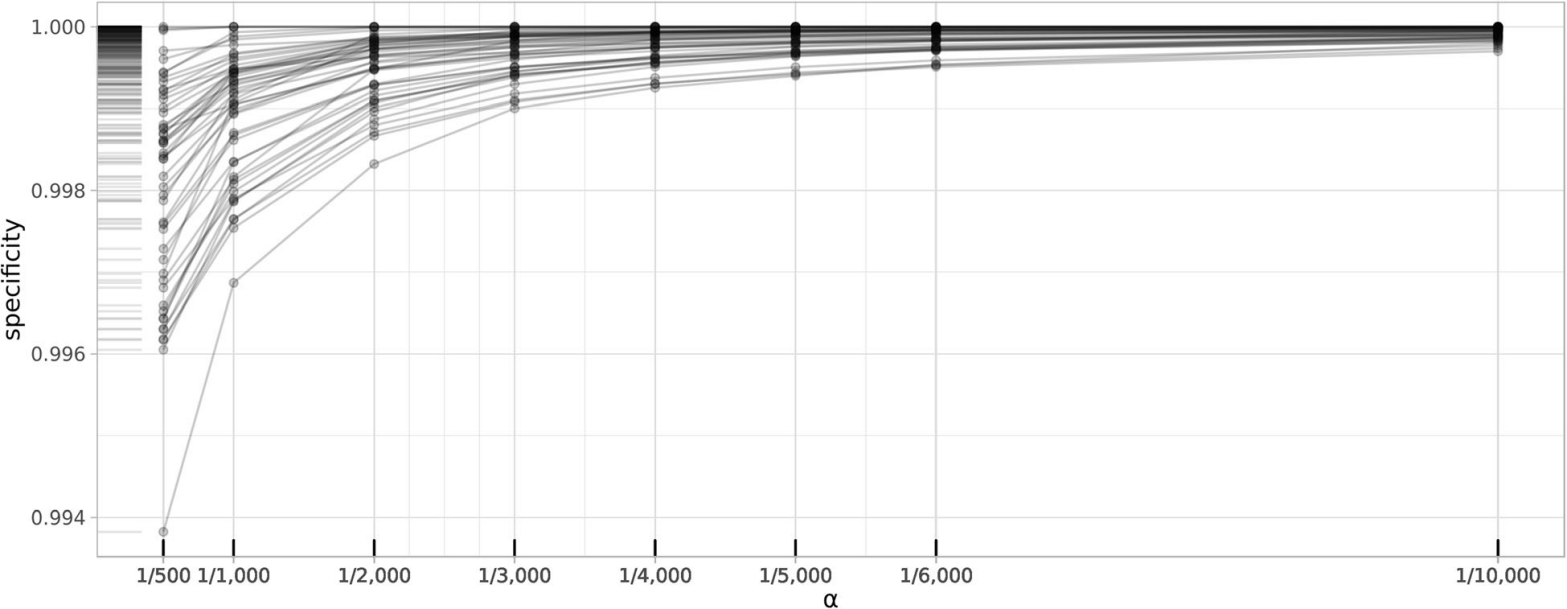
Changes in specificity of the clustering approach by ***α***

In all of the 50 lab observations, the clustering approach performed with a specificity greater than 0.9997 (Table 1). The best specificities were often obtained from the most stringent ***α*** (1/10,000), which identifies a cluster as implausible only if its population is 1/10,000 of the data subset. In this configuration, we used subsets of 10,000 data points for parallel computing. The 10,000 data points were partitioned into ***n*** clusters, and the cluster with 1 data point was identified as implausible. The lowest specificity, 0.9938, was from the most liberal configuration with ***α*** = 1/500. Overall, we found that specificity increases as ***α*** decreases (Fig. 3).

### Sensitivity

**Fig. 4:**
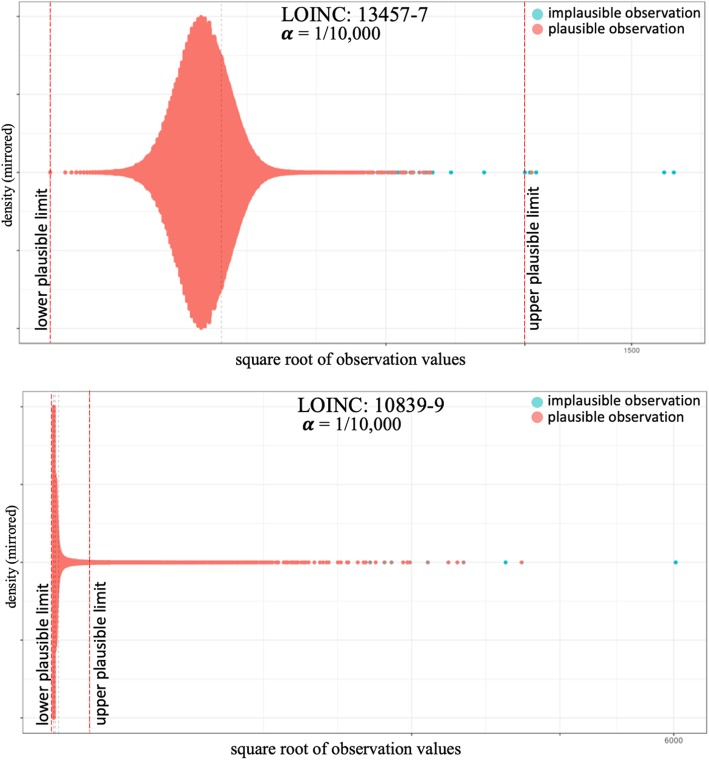
Data distribution and implausible value detection for Troponin I.cardiac (LOINC: 10839–9) and Cholesterol in LDL (LOINC: 13457–7). * x-axes are transformed to square root for visualization purpose

Sensitivity focuses on true positives, and in our case, represents how many of the implausible observations were picked up by the algorithm. Our sensitivity results we less consistent than the specificities we obtained from the clustering approach. We did not have any implausible observations in 9 of the 50 lab tests – i.e., no true positives in 18% of the labs. In the 41 remaining labs, we obtained the best performance from the most liberal configuration ***α***, where 1 in 500 data points was flagged as implausible (Table 2). It is important to evaluate the sensitivity results considering the sparsity of positives (implausible observations) in data. The number of implausible observations in the 41 labs ranged from 1 to over 39,000, representing an average of 0.0576% of the labs. Considering sparsity, a sensitivity over 0.85 would pick up most of the implausible observations. We obtained sensitivity of over 0.85 in 39 of the 41 labs that had at least 1 implausible observation. In the remaining two labs, Troponin I.cardiac (LOINC: 10839–9) and Cholesterol in LDL (LOINC: 13457–7), the best sensitivity was 0.0545 and 0.4867, respectively. Figure 4 illustrates the implausible values detected by the clustering method for the two labs. Troponin I.cardiac (LOINC: 10839–9) was an unusual case with a much higher proportion of implausible observations than expected (over 39,000 based on our silver standard implausible cutoff of [0–20] and normal range of [0.04–0.39]). Such a large number of implausible values violates our sparsity assumption under non-systematic errors – i.e., the issue should be systematic and hence is observed frequently. For Cholesterol in LDL (LOINC: 13457–7) there were over 500 positives, or implausible observations, out of over 14 million lab records.

### Comparing the clustering approach with conventional anomaly detection

**Fig. 5:**
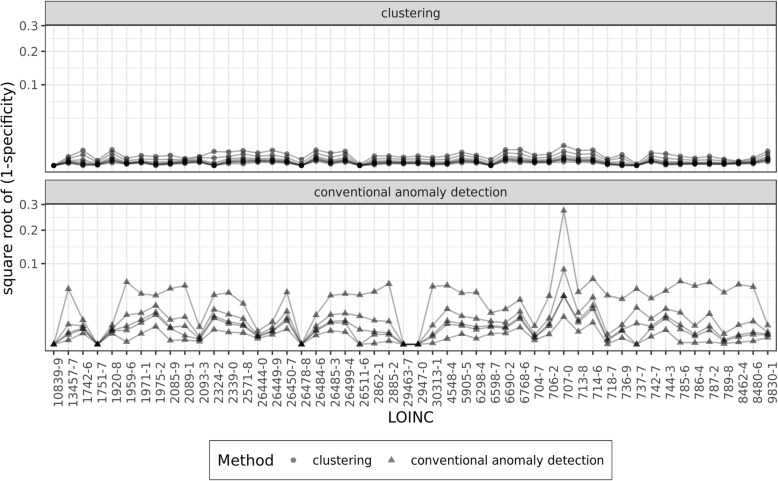
Comparing the specificity (1-specificity) performance between conventional anomaly detection and the clustering approach. * Y-axis is transformed into square root to highlight differences

The comparison of our proposed clustering approach to conventional anomaly detection (CAD) methods can be seen in Fig. 5. Overall, Fig. 5 shows that the clustering approach produced overwhelmingly better specificity than conventional anomaly detection. Best specificity from CAD methods was obtained from using 6 standard deviations as threshold for identifying outliers, which was outperformed by the clustering approach. It is important to notice that small differences among clustering results are meaningful as we deal with very large datasets for different observation types. For example, for a lab test with 10 million observations, a 0.001 difference in specificity between the clustering and CAD approaches means 10,000 more flagged observations that may need to be reviewed for plausibility.

**Fig. 6:**
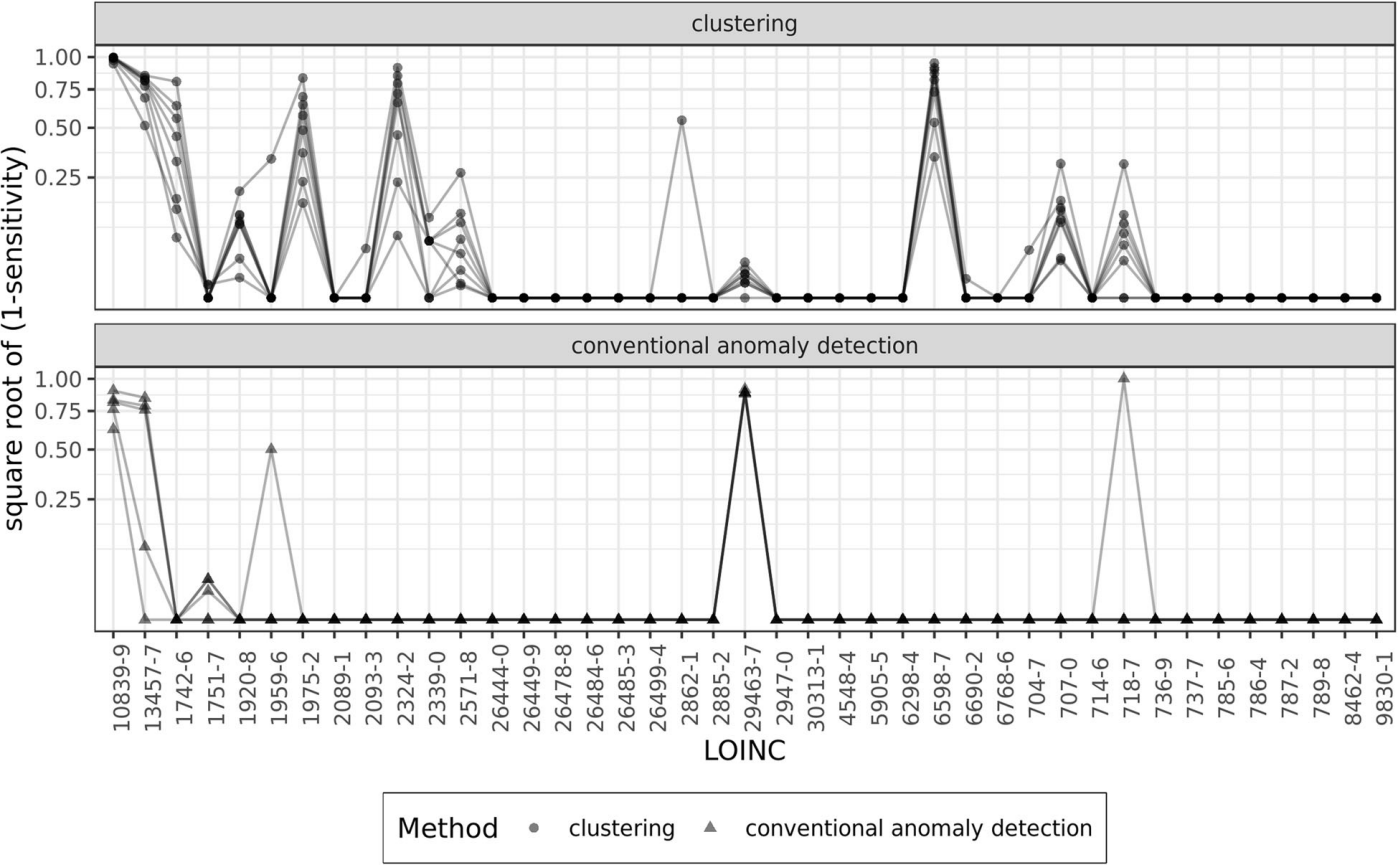
Comparing the sensitivity (1-sensitivity) performance between conventional anomaly detection and the clustering approach. * Y-axis is transformed into square root to highlight differences. 1-sensitivity is used for visualization purpose

In 31 of the 41 labs, the clustering and CAD produced similar sensitivity (Fig. 6). Among CAD methods, the best sensitivity was obtained from applying Mahalanobis Distances and 3.717526 (sqrt of 13.82) as critical value. The conventional anomaly detection (CAD) produced better sensitivity in 9 of the 41 labs for which we had implausible observations. The two largest delta in sensitivity was for Troponin T.cardiac (LOINC: 6598–7), where the clustering approach outperformed the best CAD result by 0.8794, and for Cholesterol in LDL (LOINC: 13457–7), where the best CAD result improved sensitivity by 0.5133. Outside these two labs, the average improvement in sensitivity in 9 labs was 0.1228, which considering the sparsity of implausible observations is minor.

**Fig. 7:**
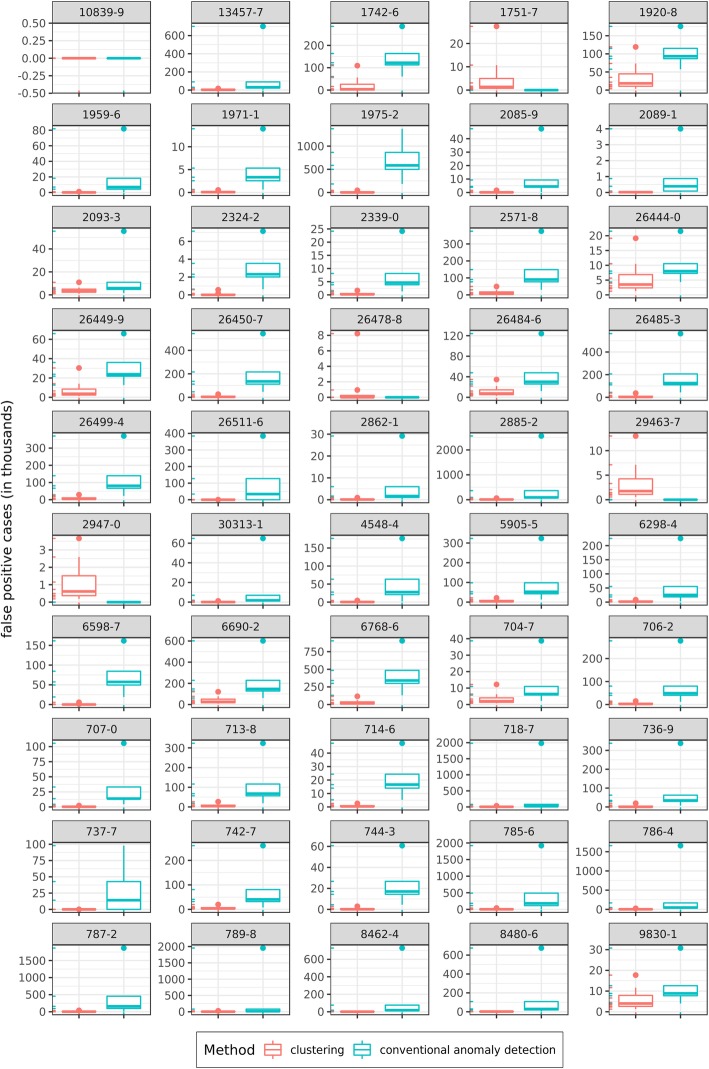
Pairwise comparison of false positive cases between conventional anomaly detection (CAD) and the clustering approaches

We further evaluated the differences between CAD methods and our proposed clustering approach through number of false positive cases in each of the labs. As discussed earlier, our goal was to minimize the frequency of observations falsely identified as implausible. Figure 7 illustrates a pairwise comparison of number of false positive cases identified by each approach for each of the 50 lab observations. In 45 out of 50 labs (90%), the clustering approach produced a statistically significant smaller number of false positive cases.

More importantly, when the clustering approach outperformed the CAD approach, the gaps between the two approaches signified a large number of false positives. As the Y-axes show (scale is transformed in thousands) the conventional anomaly detection often identifies thousands of more plausible observations as implausible, compared with the clustering approach.

## Discussion

EHRs provide massive amounts of observational data. Biological data have certain properties that are distinct in their distribution from other types of the so called “Big Data.” We designed, implemented, and tested an unsupervised clustering approach for identifying implausible records in clinical observation data. Our approach is based on two linked hypotheses that 1) if no systematic data entry errors exist, implausible clinical observations in electronic health records are sparse, and therefore 2) if clustered appropriately, clusters with very small populations should represent implausible observations. Using EHR laboratory results data from Partners Healthcare, our results supported both hypotheses. A set of plots on selected labs are available in Additional file 1: Figure S1.

We also demonstrated that the clustering approach outperforms conventional anomaly detection (CAD) approaches in identifying implausible lab observations. In biological data, an outlier may or may not be implausible. For example, if an EHR has a patient record for a 121-year-old woman, the record is very likely an outlier, but not biologically implausible – according to the list of the verified oldest people in 1997, a French woman died at the purported age of 122 years, 164 days. Our results showed that, through the sparsity assumption (***α***) the clustering approach can improve differentiating the implausibles from the outliers, in comparison with the conventional methods. In anomaly detection, outliers are often conceived as extreme values at the two tails of the distribution. Our approach expands the conventional definition of an outlier, by searching for observations that are sparse considering their value and the density of the cluster they belong to, relative to the rest of the data points. Such observations can be found anywhere across the distribution of data – i.e., implausible values can still be extremely high or low, or just different enough from the rest of data points. For example, on a post-partum unit where ages may be ~ 0–4 days old or ~ 14–45 years old, one would not expect anyone 6 years old. In the 50 observation types we evaluated in this study, we did not find implausible values outsides of the upper or lower tails of the data distribution. However, labs and vital signs can be evaluated as multidimensional data by including various dimensions such as patients’ demographics and comorbidities, in which case a proper implementation of the clustering method can also detect and flag other types of implausible records.

The clustering approach for identifying implausible observations offers a precise “Big Data” solution for clinical and biological observations stored in electronic medical records. The clustering process is observation-specific, as the number of clusters and partitioning is specified for each group of observations. As a result, it produced low false positives – plausible observations mistakenly identified as implausible. In contrast, we showed that CAD approaches produce a high number of false positives. This difference is a huge benefit for the clustering approach from an informatics standpoint regarding implementation in large scale data repositories.

### Limitations and directions for future research

We used a hybrid hierarchical K-means algorithm, HK-means, because K-means algorithms are generally sensitive to outliers and the hybrid method improves K-means’ reliance on the random centroid initialization. We can imagine that other distance-based or density-based unsupervised clustering algorithms might be also effective in identifying rare implausible clinical observations.

Many of clinical observations stored in EHR data are unlabeled. Unsupervised learning approaches offer many promising solutions for patient characterization. Nevertheless, these approaches require specification of several hyper-parameter. In our case, performance of the clustering approach depended on the number of clusters, initial random assignment of cluster centroids, and the threshold for flagging clusters as implausible. In addition, implementation of the approach on very large datasets was challenging. To address some of these challenges, we had to be creative in selecting the clustering algorithm, applying feature transformations, and developing the *kluster* procedure [28] to approximate the number of clusters, all of which would be practical for future unsupervised learning efforts that aim to ascertain meaningful patient sub-groups.

We randomly shuffled the data to prevent any potential sorting in breaking the data to folds for parallel computing. Further research is needed to ascertain whether some systematic approach to parallelization (e.g., breaking the data by age and gender) can improve the unsupervised implausible observation detection results.

Moreover, we have tested the clustering approach against 50 EHR observations. This demonstration was limited to a small set of observations due to the need for silver-standards to measure sensitivity and specificity of our proposed approach. However, we encourage the readers to envision further applications of this approach to other clinical observations, as well as complex combinations of observations in multiple dimensions, for which specification of manual silver-standards are virtually implausible.

Finally, the primary use case for this method is intended for an algorithmic screening of EHR laboratory observations for potential implausible values that would not be suggested for secondary use. The methodology can be applied to other use cases, such as identifying exceptionally well-performing clinicians or detecting unusual patient management actions, would require identification of outlying but still physiologically plausible values. However, because the evaluation criteria may be different for other use cases (we focused on false positives for the detection of implausible observations in large scale EHR repositories), further work may be needed to adjust the ***α*** in order to optimize performance. For instance, in cases where outliers are more numerous and/or are closer to the remainder of the data, chart review would allow a more definitive (although not always completely definitive) determination of whether a value is clearly erroneous or not.

### Implementation considerations

The algorithmic solution we presented in this paper proved as a feasible alternative for replacing the current manual rule-based procedures for identifying biologically implausible values. Given the size of data and the emphasis on sensitivity versus specificity, the choice of ***α*** (the ratio for flagging a cluster as implausible) can vary. In large clinical data repositories, an ***α*** between 1/4000 and 1/6000 would provide good balance between true positives and false negatives. A smaller ***α*** is also computationally more expensive. When the size of the dataset is small, a small ***α*** will be more appropriate. Nevertheless, the value for ***α*** can be adjusted over time to address the institutional needs and comfort level. Due to superb specificity, implementing the clustering approach offers a low-risk solution to an expensive manual procedure that is hard to implement. We envision the proposed solution to constantly operate on the data base servers where EHR data are stored. We recommend that our algorithmic solution should be used to augment (rather than replace) human decision-making for improving quality of EHR data. After the implausible values are detected and flagged, a workflow is needed to initiate further actions needed in order to determine the density of the flagged observations. As we showed, because the flagged records are also sparse, the frequency of such flags should not be of concern.

For a specific lab test (Troponin I.cardiac – LOINC: 10839–9) we found an unusual high number of implausible observations (over 39,000), which was calculated based on our silver standard implausible cutoff of [0–20] and normal range of [0.04–0.39]. Further work is needed to fully discern the root cause for this issue in the data. Nearly all of these implausible values originated from a single type of Cardiac Troponin I test run at Brigham and Women’s hospital between 2001 and 2008. Although RPDR tries to normalize the units among labs assigned to the same LOINC code, it is possible that this particular cTI test is being reported with different units than the other tests in this LOINC code (for example, 0.41 could be reported routinely as 41). We are presently in discussion with the RPDR team about this possibility. Nonetheless, even in this case, all of the clustering algorithms produced results with 100% specificity, meaning that even when the sparsity assumption is not fulfilled, false positive cases will not be introduced.

## Conclusion

Detecting implausible clinical observations in Electronic Health Record (EHR) data is a challenge, requiring availability of standards thresholds and rule-based procedures to query observations that are out of the plausibility range. Establishing rule-based procedures to address this task entails extensive hard-coding that would accumulate over time and dimensionality. We proposed an alternative viable algorithmic solution, using unsupervised clustering approach. The clustering approach is superior than conventional anomaly detection approaches and adaptable to different types of numerical EHR observation data.

### Additional file

Additional file 1: **Figure S1.** Data distribution and implausible value detection for a set of selected EHR observation types.* x-axes are transformed to square root for visualization purpose. (PDF 369 kb)

## Data Availability

Since access to this dataset requires consent from IRB, the dataset cannot be made available publicly or upon request.

## Appendix

**Table 4:** Silver-standard low and high ranges for implausible observation values

LOINC	Low implausible	High implausible	Long common name
1742-6	0	2500	Alanine aminotransferase [Enzymatic activity/volume] in Serum or Plasma
1751-7	0	20	Albumin [Mass/volume] in Serum or Plasma
2862-1	0	20	Albumin [Mass/volume] in Serum or Plasma by Electrophoresis
6768-6	0	5000	Alkaline phosphatase [Enzymatic activity/volume] in Serum or Plasma
1920-8	0	12500	Aspartate aminotransferase [Enzymatic activity/volume] in Serum or Plasma
26444-0	0	100	Basophils [#/volume] in Blood
704-7	0	5	Basophils [#/volume] in Blood by Automated count
706-2	0	50	Basophils/100 leukocytes in Blood by Automated count
707-0	0	50	Basophils/100 leukocytes in Blood by Manual count
1959-6	0	100	Bicarbonate [Moles/volume] in Blood
1971-1	0	50	Bilirubin.indirect [Mass/volume] in Serum or Plasma
1975-2	0	50	Bilirubin.total [Mass/volume] in Serum or Plasma
29463-7	0	1400	Body weight
2093-3	0	1500	Cholesterol [Mass/volume] in Serum or Plasma
2085-9	0	450	Cholesterol in HDL [Mass/volume] in Serum or Plasma
2089-1	0	780	Cholesterol in LDL [Mass/volume] in Serum or Plasma
13457-7	0	1000	Cholesterol in LDL [Mass/volume] in Serum or Plasma by calculation
9830-1	0	100	Cholesterol.total/Cholesterol in HDL [Mass Ratio] in Serum or Plasma
8462-4	0	360	Diastolic blood pressure
26449-9	0	100	Eosinophils [#/volume] in Blood
26450-7	0	100	Eosinophils/100 leukocytes in Blood
713-8	0	100	Eosinophils/100 leukocytes in Blood by Automated count
714-6	0	100	Eosinophils/100 leukocytes in Blood by Manual count
789-8	0	25	Erythrocytes [#/volume] in Blood by Automated count
2324-2	0	2500	Gamma glutamyl transferase [Enzymatic activity/volume] in Serum or Plasma
2339-0	0	1000	Glucose [Mass/volume] in Blood
30313-1	0.5	75	Hemoglobin [Mass/volume] in Arterial blood
718-7	0.5	75	Hemoglobin [Mass/volume] in Blood
4548-4	0	30	Hemoglobin A1c/Hemoglobin.total in Blood
6690-2	0	500	Leukocytes [#/volume] in Blood by Automated count
26478-8	0	100	Lymphocytes/100 leukocytes in Blood
736-9	0	100	Lymphocytes/100 leukocytes in Blood by Automated count
737-7	0	100	Lymphocytes/100 leukocytes in Blood by Manual count
785-6	0	100	MCH [Entitic mass] by Automated count
786-4	0	100	MCHC [Mass/volume] by Automated count
787-2	0	400	MCV [Entitic volume] by Automated count
26484-6	0	100	Monocytes [#/volume] in Blood
742-7	0	100	Monocytes [#/volume] in Blood by Automated count
26485-3	0	100	Monocytes/100 leukocytes in Blood
5905-5	0	100	Monocytes/100 leukocytes in Blood by Automated count
744-3	0	100	Monocytes/100 leukocytes in Blood by Manual count
26499-4	0	400	Neutrophils [#/volume] in Blood
26511-6	0	100	Neutrophils/100 leukocytes in Blood
6298-4	0	30	Potassium [Moles/volume] in Blood
2885-2	0	30	Protein [Mass/volume] in Serum or Plasma
2947-0	0	580	Sodium [Moles/volume] in Blood
8480-6	0	560	Systolic blood pressure
2571-8	0	2500	Triglyceride [Mass/volume] in Serum or Plasma
10839-9	0	20	Troponin I.cardiac [Mass/volume] in Serum or Plasma
6598-7	0	20	Troponin T.cardiac [Mass/volume] in Serum or Plasma

^a^the silver standard low and high ranges for implausible observation values are defined by the authors based on literature search and expert judgement, and validate using data distributions

## References

[1] Brown JS, Kahn M, Toh S (2013). Data quality assessment for comparative effectiveness research in distributed data networks. Med Care.

[2] Weiskopf NG, Weng C (2013). Methods and dimensions of electronic health record data quality assessment: enabling reuse for clinical research. J Am Med Inform Assoc [Internet].

[3] Kahn Michael G., Callahan Tiffany J., Barnard Juliana, Bauck Alan E., Brown Jeff, Davidson Bruce N., Estiri Hossein, Goerg Carsten, Holve Erin, Johnson Steven G., Liaw Siaw-Teng, Hamilton-Lopez Marianne, Meeker Daniella, Ong Toan C., Ryan Patrick, Shang Ning, Weiskopf Nicole G., Weng Chunhua, Zozus Meredith N., Schilling Lisa (2016). A Harmonized Data Quality Assessment Terminology and Framework for the Secondary Use of Electronic Health Record Data. eGEMs (Generating Evidence & Methods to improve patient outcomes).

[4] Ghahramani Z. Unsupervised Learning. In: Bousquet O, von Luxburg U, Rätsch G, editors. Advanced Lectures on Machine Learning. ML 2003. Lecture Notes in Computer Science, vol 3176. Berlin, Heidelberg: Springer; 2004.

[5] Hauskrecht M, Batal I, Hong C, Nguyen Q, Cooper GF, Visweswaran S (2016). Outlier-based detection of unusual patient-management actions: an ICU study. J Biomed Inform.

[6] Bouarfa L, Dankelman J (2012). Workflow mining and outlier detection from clinical activity logs. J Biomed Inform.

[7] Presbitero Alva, Quax Rick, Krzhizhanovskaya Valeria, Sloot Peter (2017). Anomaly Detection in Clinical Data of Patients Undergoing Heart Surgery. Procedia Computer Science.

[8] Antonelli Dario, Bruno Giulia, Chiusano Silvia (2013). Anomaly detection in medical treatment to discover unusual patient management. IIE Transactions on Healthcare Systems Engineering.

[9] Ray S, Wright A. Detecting anomalies in alert firing within clinical decision support systems using anomaly/outlier detection techniques. Proc. 7th ACM Int. Conf. Bioinformatics, Comput. Biol. Heal. Informatics. New York: ACM; 2016. p. 185–90. Available from: http://doi.acm.org/10.1145/2975167.2975186

[10] Ray S, McEvoy DS, Aaron S, Hickman TT, Wright A (2018). Using statistical anomaly detection models to find clinical decision support malfunctions. J Am Med Informatics Assoc.

[11] Wilson B, Tseng CL, Soroka O, Pogach LM, Aron DC (2017). Identification of outliers and positive deviants for healthcare improvement: looking for high performers in hypoglycemia safety in patients with diabetes. BMC Health Serv Res.

[12] Deneshkumar V, Senthamaraikannan K, Manikandan M. Identification of outliers in medical diagnostic system using data mining techniques. Int J Stat Appl. 2014;4(6):241–8.

[13] Chandola Varun, Banerjee Arindam, Kumar Vipin (2009). Anomaly detection. ACM Computing Surveys.

[14] Hodge Victoria J., Austin Jim (2004). A Survey of Outlier Detection Methodologies. Artificial Intelligence Review.

[15] Aggarwal Charu C., Yu Philip S. (2001). Outlier detection for high dimensional data. ACM SIGMOD Record.

[16] Knorr Edwin M., Ng Raymond T., Tucakov Vladimir (2000). Distance-based outliers: algorithms and applications. The VLDB Journal The International Journal on Very Large Data Bases.

[17] Ben-Gal I. Outlier Detection. In: Maimon O, Rokach L, editors. Data Mining and Knowledge Discovery Handbook. Boston: Springer; 2005.

[18] Gaspar J, Catumbela E, Marques B, Freitas A. A systematic review of outliers detection techniques in medical data - preliminary study. Heal. 2011. Proc Int Conf Heal Informatics. 2011.

[19] Hastie T, Tibshirani R, Friedman J. The elements of statistical learning: data mining, inference, and prediction: Springer Ser. Stat; 2009.

[20] Jain AK (2010). Data clustering: 50 years beyond K-means. Pattern Recogn Lett.

[21] MacQueen J. Some methods for classification and analysis of multivariate observations. Proc. Fifth Berkeley Symp. Math. Stat. Probab. Vol. 1 Stat. Berkeley, Calif.: University of California Press; 1967. p. 281–97. Available from: http://projecteuclid.org/euclid.bsmsp/1200512992.

[22] Chawla S, Gionis A (2013). *k* -means–: a unified approach to clustering and outlier detection. Proc. 2013 SIAM Int. Conf. Data min.

[23] Chen B, Tai PC, Harrison R, Pan Y (2005). Novel hybrid hierarchical-K-means clustering method (H-K-means) for microarray analysis. IEEE Comput Syst Bioinforma Conf Work Poster Abstr.

[24] Sugar CA, James GM (2003). Finding the number of clusters in a dataset. J. Am. Stat. Assoc.

[25] Hamerly G, Elkan C. Learning the k in k means. Adv neural Inf Process. 2004;17:1–8. Available from: books.nips.cc/papers/files/nips16/NIPS2003_AA36.pdf%5Cnhttp://books.google.com/books?hl=en&lr=&id=0F-9C7K8fQ8C&oi=fnd&pg=PA281&dq=Learning+the+k+in+k-means&ots=TGLvqYQa40&sig=SDu4cZ9TCeU8a5MoG1uMcRLQGFE.

[26] Fraley C, Raftery AE (2002). Model-based clustering, discriminant analysis, and density estimation. J Am Stat Assoc.

[27] Nalichowski R, Keogh D, Chueh HC, Murphy SN. Calculating the benefits of a research patient data repository. AMIA Annu Symp Proc United States. 2006. p. 1044. PMC1839563 17238663

[28] Estiri H, Omran BA, Murphy SN (2018). Kluster : an efficient scalable procedure for approximating the number of clusters in unsupervised learning. Big Data Res.

[29] De Maesschalck R, Jouan-Rimbaud D, Massart DLL. The Mahalanobis distance. Chemom Intell Lab Syst. 2000;50:1–18.

[30] Filzmoser P. A multivariate outlier detection method. Seventh Int Conf Comput Data Anal Model. 2004.

