# Supplementary material for: A clustering approach for detecting implausible observation values in electronic health records data
Source: BMC Med Inform Decis Mak. 2019 Jul 23;19:142. doi: 10.1186/s12911-019-0852-6 (PMC6652024; doi:10.1186/s12911-019-0852-6)

Appendix

Data distribution and implausible value detection for a set of selected EHR observation types.

* x-axes are transformed to square root for visualization purpose.

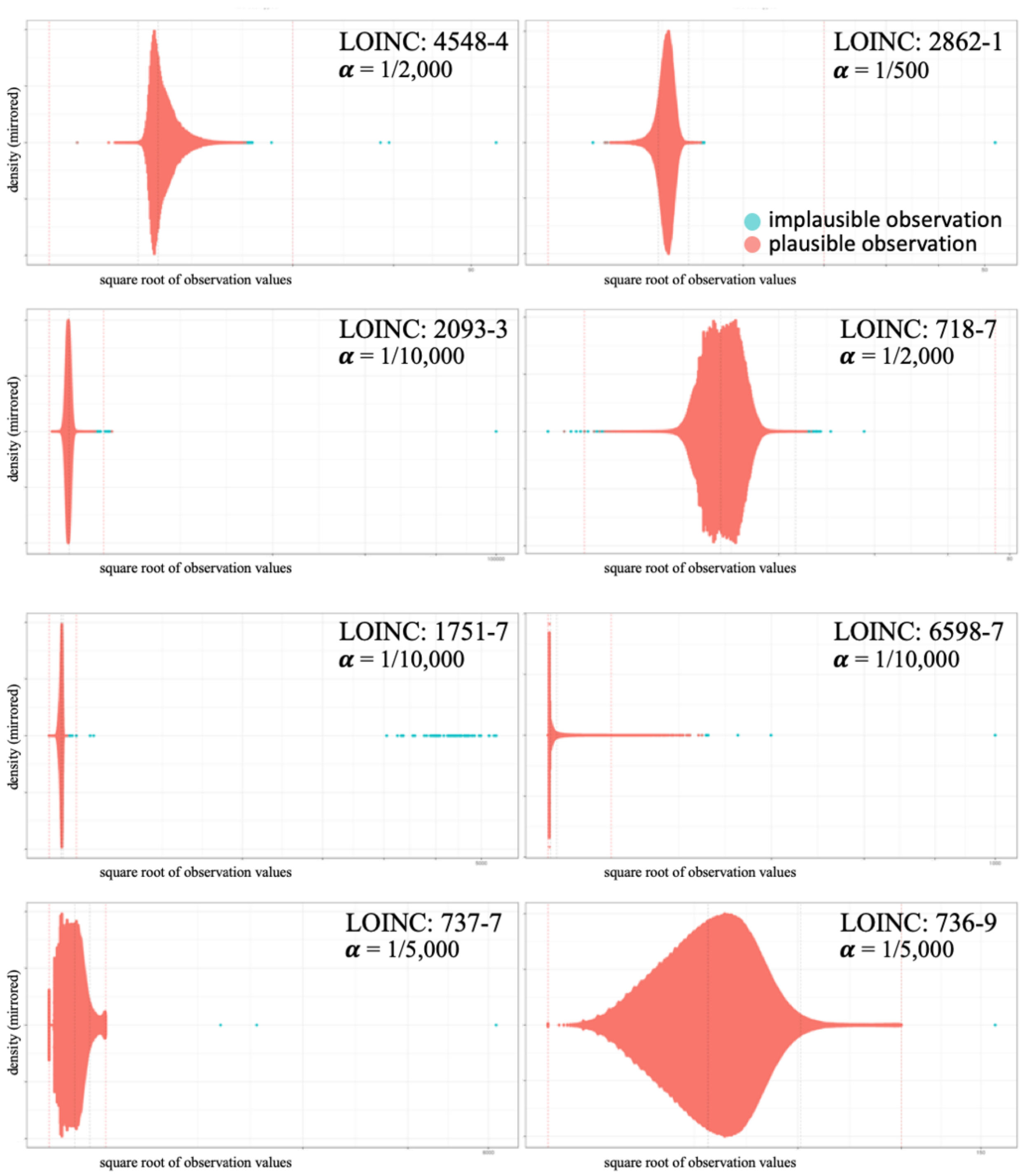

Supplement: Supplementary file 1 — Figure S1. Data distribution and implausible value detection for a set of selected EHR observation types.* x-axes are transformed to square root for visualization purpose. (PDF 369 kb) [file 12911_2019_852_MOESM1_ESM.pdf]
